# Individual and contextual characteristics associated with concurrent risk factors among adolescents: a multilevel cross-sectional study based on the 2019 Brazilian National Health Survey

**DOI:** 10.1590/S2237-96222025v34e20240453.en

**Published:** 2025-09-08

**Authors:** Ruàn Éverton de Souza Silva, Eduardo Araujo Lima, Antonio Valdeir Lopes da Silva, Shelda Santos Silva, Jéssica Fernanda de Sousa, Edina Araújo Rodrigues Oliveira, Danilla Michelle Costa e Silva, Mailson Fontes de Carvalho, Rumão Batista Nunes de Carvalho

**Affiliations:** 1 Universidade Federal do Piauí, Picos, PI, Brazil; 2 Universidade Federal do Recôncavo da Bahia, Salvador, BA, Brazil

**Keywords:** Risk Factors, Socioeconomic Factors, Health Inequalities, Adolescent, Cross-Sectional Studies, Factores de Riesgo, Factores Socioeconómicos, Desigualdades en Salud, Adolescente, Estudios Transversales

## Abstract

**Objective:**

To assess the simultaneity of risk behaviors for chronic non-communicable diseases and their association with individual and contextual characteristics in Brazilian adolescents.

**Methods:**

Cross-sectional study using data from the 2019 Brazilian National Health Survey. The simultaneity of factors of the consumption of ultra-processed foods, level of physical activity, smoking and alcohol use was analyzed, according to individual and contextual characteristics, estimating the odds ratios (OR) and respective 95% confidence intervals (95%CI) for fixed effects and variance and 95%CI for random effects, through multilevel polytomous logistic regression.

**Results:**

Among the 4,336 adolescents evaluated, the most prevalent combination of two risk behaviors included the consumption of ultra-processed foods and insufficient level of physical activity (13.3%; 95%CI 11.6; 15.3). The most frequent combination of three factors included these behaviors combined with habitual alcohol use (3.6%; 95%CI 2.7; 4.8). The chances of combinations of three or four factors were lower in females (OR 0.44; 95%CI 0.32; 0.60) and in rural areas (OR 0.46; 95%CI 0.31; 0.70) and higher in older adolescents (OR 2.57; 95%CI 1.72; 3.83), with higher levels of education (OR 8.11; 95%CI 2.41; 27.26) and living without partners (OR 3.83; 95%CI 1.10; 13.35). High Human Development Indices increased these chances (OR 7.28; 95%CI 3.81; 13.92), while high Social Vulnerability Indices (OR 0.25; 95%CI 0.11; 0.58) and Gini Indices (OR 0.28; 95%CI 0.15; 0.52) reduced them.

**Conclusion:**

The occurrence of multiple risk behaviors in adolescents is more likely among older, male, single and more educated adolescents, especially in areas of greater socioeconomic development.

## Resumen

**Objetivo:**

Evaluar la simultaneidad de comportamientos de riesgo para enfermedades crónicas no transmisibles y su asociación con características individuales y contextuales en adolescentes brasileños.

**Métodos:**

Estudio transversal, con datos de la Encuesta Nacional de Salud, 2019. Se analizó la simultaneidad de los factores consumo de alimentos ultraprocesados, nivel de actividad física, tabaquismo y consumo de alcohol, según características individuales y contextuales, estimando las razones de probabilidades (odds ratio - OR) y respectivos intervalos de confianza del 95% (IC95%) para efectos fijos y varianza e IC95% para efectos aleatorios, mediante regresión logística politómica multinivel.

**Resultados:**

Entre los 4.336 adolescentes evaluados, la combinación más prevalente de dos conductas de riesgo fue el consumo de alimentos ultraprocesados y el nivel insuficiente de actividad física (13,3%; IC95% 11,6; 15,3). La combinación más frecuente de tres factores incluyó estos comportamientos combinados con el consumo habitual de alcohol (3,6%; IC95% 2,7; 4,8). Las probabilidades de combinaciones de tres o cuatro factores fueron menores en mujeres (OR0,44; IC95% 0,32; 0,60) y en zonas rurales (OR 0,46; IC95% 0,31; 0,70) y mayores en adolescentes mayores (OR 2,57; IC95% 1,72;3,83), con niveles superiores de educación (OR 8,11; IC95% 2,41; 27,26) y que vivían sin pareja (OR 3,83; IC95% 1,10;13,35). Los altos Índices de Desarrollo Humano aumentaron estas probabilidades (OR 7,28; IC95% 3,81; 13,92), mientras que los altos Índices de Vulnerabilidad Social (OR 0,25; IC95% 0,11; 0,58) y los Índices de Gini (OR 0,28; IC95% 0,15; 0,52) las redujeron.

**Conclusión:**

La ocurrencia de múltiples conductas de riesgo en adolescentes es más probable entre adolescentes mayores, varones, solteros y con mayor nivel educativo, especialmente en áreas de mayor desarrollo socioeconómico.

**Ethical aspects**

This research used public domain data and anonymized databases.

## Introduction

Chronic non-communicable diseases represent a major public health problem worldwide and are associated with significant impacts on the economy of families and society (1). In Brazil, the impact of these diseases highlights a growing crisis in the health system, being responsible for almost 70.0% of premature mortality in the country (2).

Adolescence, a transitional stage between childhood and adulthood, is marked by intense physical, emotional, and social changes that play a vital role in the adoption of risky behaviors, such as poor diet, smoking, a sedentary lifestyle, and alcohol consumption (3). This phase is characterized by psychological vulnerability, the search for acceptance and autonomy, making young people more susceptible to experimenting with practices that are harmful to their health (3,4).

Among young people aged 10 to 20, physical inactivity and sedentary behavior, especially resulting from high exposure to screens, are linked to increased obesity, low consumption of fruits and vegetables, excessive consumption of salt and sugary drinks, depression, and reduced quality of life (4,5). These behaviors, when fixed at this stage, are related to approximately two-thirds of premature deaths in adults (5).

Individual characteristics such as sex, age, race/skin color and maternal education are strongly associated with risk behaviors (6) and reinforce the importance of monitoring these factors, aiming not only at monitoring priority groups, but also at promoting the health of exposed individuals (7). Contextual aspects can also directly influence risk behaviors for chronic non-communicable diseases. Quality health promotion programs (8), school attendance, and parental support (9) help reduce these risk behaviors. Conversely, inequities such as low levels of education, difficulties in accessing the job market and poor income distribution contribute to the prevalence of these diseases (10).

Despite the significant impact of individual and contextual factors on the adoption of risk behaviors for chronic non-communicable diseases, there is still little research analyzing the simultaneous manifestation of these factors, considering the associated contexts (8,9). This study aims to evaluate the simultaneity of risk behaviors for chronic non-communicable diseases and associate them with individual and contextual characteristics in Brazilian adolescents.

## Methods

### Study design

This is a cross-sectional population-based study, using data from the 2019 Brazilian National Health Survey (PNS). 

### Context

The 2019 PNS, carried out by the Brazilian Institute of Geography and Statistics (IBGE) in partnership with the Ministry of Health, is a representative survey of the Brazilian population, which collected data on determinants and health needs of Brazilians aged 15 or over, living in permanent households, using cluster sampling in three stages: stratification of primary sampling units (census sectors with probability proportional to the number of households), random selection of households and, finally, random selection of a resident (11).

### Participants 

This study used data from adolescents aged 15 to 19 (12), living in permanent private homes in all Federative Units of the country. 

### Variables 

Individual and contextual variables were used to analyze the outcome, which was the simultaneity of health risk factors. Individual risk behaviors considered included consumption of ultra-processed foods, insufficient level of physical activity during free time, smoking, and habitual alcohol use. Individual characteristics were also assessed, such as: sex (male; female), age in years (15 to 17; 18 to 19), race/skin color (white; non-white), education (no education; incomplete elementary school; complete elementary school; complete high school; complete higher education), marital status (with a partner; without a partner), area of residence (urban; rural), perception of poor health (no; yes) and health insurance coverage (no; yes).

Regarding contextual characteristics, the following equity indicators were used: Municipal Human Development Index (medium; high; very high) Social Vulnerability Index (very low; low; medium) and the Gini Index (1st tertile; 2nd tertile; 3rd tertile). 

### Data sources and measurement 

The data for the analysis of individual variables come from the PNS 2019, which has free access through the IBGE website. This national survey collected household data, conducted by trained interviewers who applied a questionnaire containing three instruments: a) household, b) from all residents of the household and c) individual. 

The consumption of ultra-processed foods was characterized by frequent intake, defined as five or more times a week, of products such as cookies/stuffed biscuits, chocolate, gelatin, candies and similar products, soft drinks/artificial juices and snacks that replace lunch such as sandwiches, savory snacks, pizzas, hot dogs, among others (0 = no; 1 = yes). An insufficient level of physical activity during free time was considered when the individual reported not performing at least 300 minutes of physical activity per week, considering only activities performed during free time (0 = no; 1 = yes) (13). Regarding smoking and habitual alcohol use, any consumption of these indicators in the last 30 days prior to the survey was considered a risk factor (0 = no; 1 = yes), given the greater vulnerability of adolescents to the damage caused by these substances, the sale of which is prohibited for this age group in Brazil (14).

The perception of poor health was obtained by self-classifying one’s own health as “very good,” “good,” “regular,” “bad” or “very bad.” In this analysis, this classification was separated into two categories: good health (regular, good, and very good) and poor health (poor and very poor) (0 = no; 1 = yes). Health insurance coverage was assessed based on the statement about the availability of a private, company or public agency health insurance plan (0 = yes; 1 = no). 

Regarding contextual aspects, data were obtained from the Atlas of Human Development in Brazil and the Institute of Applied Economic Research. The Social Vulnerability Index is calculated from sixteen indicators grouped into three sub-indices: urban infrastructure, human capital and income and work, ranging from 0.000 to 1.000, with 0 indicating no social vulnerability and 1 representing the maximum level of vulnerability (0 to 0.200 - very low social vulnerability; 0.201 and 0.300 - low social vulnerability; 0.301 and 0.400 - medium social vulnerability; 0.401 and 0.500 - high social vulnerability, and >0.500 - very high social vulnerability) (15).

The Municipal Human Development Index also varies from 0.000 to 1.000, where the closer to 1, the greater the human development of a given geographic region. It is classified as very low (0.000-0.499), low (0.500-0.599), medium (0.600-0.699), high (0.700-0.799) and very high (0.800-1.000) (16).

The Gini Index is used as a parameter to measure income inequality among individuals in a population, being quantified between 0 and 1, where values closer to 0 indicate less income inequality, and values closer to 1 indicate greater inequality (17).

In this study, no values were observed for the low and very low classifications for the Municipal Human Development Index and high and very high for the Social Vulnerability Index. 

### Study size

The sample size of the 2019 PNS considered the desired precision to estimate the main risk and protection factors for chronic non-communicable diseases, disease prevalence, and access to health services, among other variables. The expected sample was 108,525 households, and data were collected from 94,114, with a non-response rate of 6.4% (18). 90,846 individuals (11) were interviewed, of which 4,336 were adolescents between 15 and 19 years old, who made up this analysis. 

### Statistical methods

Initially, a descriptive analysis was conducted with simple and relative frequencies of the sociodemographic characteristics of the adolescents (Table 1). Furthermore, the exclusive occurrence of all possible combinations between the four risk factors was analyzed, including the absence of factors and their respective confidence intervals (Table 2).

**Table 1:** Proportions and 95% confidence intervals (95%CI) of adolescents according to sociodemographic characteristics. Brasil, 2019 (n=4.336)

Variables	Prevalence (%)	(95%CI)
Gender		
Male	50,0	(47.2 ;52.7)
Female	50,0	(47.3; 52.8)
**Age** (years)		
15-17	59.7	(57.1; 62.3)
18-19	40.3	(37.7; 42.9)
**Race/skin solor**		
White	37.4	(34.6; 40.3)
Non-white	62.6	(59.7; 65.4)
Education		
No schooling	1.3	(0.7; 2.5)
Incomplete elementary school	18.7	(16.7; 21.0)
Completed elementary education	55.0	(52.3; 57.8)
Completed high school	24.7	(22.5; 27.2)
Completed higher education	0.1	(0.0; 0.5)
**Marital status**		
With a partner	2.3	(1.7; 3.0)
Without a partner	97.7	(97.0; 98.3)
Urban		
Area	83.6	(81.5; 85.5)
Rural	16.4	(14.5; 18.5)
**Poor health perception**		
No	97.8	(96.9; 98.5)
Yes	2.2	(1.5; 3.1)
**Health insurance**		
Yes	18.1	(16.1; 20.3)
No	81.9	(79.7; 83.9)

**Table 2:** Proportions and 95% confidence intervals (95%CI) of combinations of health risk behaviors among adolescents. Brasil, 2019 (n=4.336)

Risk factors	Ultra-processed foods	Smoking	Physical inactivity	Habitual alcohol use	Prevalence (95%CI)
0	-	-	-	-	25.6 (23.3; 28.0)
1	-	-	-	+	4.6 (3.5; 6.0)
1	-	-	+	-	15.4 (13.6; 17.4)
1	-	+	-	-	0.6 (0.3; 0.9)
1	+	-	-	-	23.6 (21.4; 26.0)
2	-	-	+	+	3.1 (2.4; 4.1)
2	-	+	-	+	1,2 (0.7; 2.1)
2	-	+	+	-	0.2 (0.1; 0.7)
2	+	-	-	+	4.6 (3.3; 6.3)
2	+	-	+	-	13.3 (11.6; 15.3)
2	+	+	-	-	1.0 (0.6; 1.5)
3	-	+	+	+	0.4 (0.2; 0.8)
3	+	-	+	+	3.6 (2.7; 4.8)
3	+	+	-	+	1.8 (1.0; 3.2)
3	+	+	+	-	0.3 (0.2; 0.5)
4	+	+	+	+	0.7 (0.4; 1.2)

In complex samples, sampling weight reflects the probability of selection in clusters in hierarchical samples, differing from frequency samples (19). Failure to scale these weights may bias the estimates. Therefore, it was decided to adjust them only at the first level, since at the second level it does not significantly affect the parameter estimates and their standard errors (20). 

As the sample studied presents small strata without the sampling plan, the weight scaling formula was applied, comprised of wij*=wij(nj/∑wij) (19,20), where wij* represents the scaled weight for individual i in state j; wij symbolizes the unscaled weight for individual i in state j; and nj is the number of sampling in state j. The stratum variable was used to indicate the grouping structure, while the household weight variable with non-interview correction without calibration by population projection constituted the individual sampling weight (19,20).

The estimate of prevalence and respective 95% confidence interval of the simultaneity of risk behaviors and of each risk factor were done independently. Multilevel polytomous logistic regression was also conducted, using the Logit multinomial (mlogit) model, by applying generalized structural equation modeling (gsem) to investigate the association between the simultaneity of risk factors (1, 2, 3/4 risk factors) and individual and contextual variables. The odds ratios (OR) and respective 95% confidence intervals (95%CI) for fixed effects and variance and 95%CI for random effects were estimated (Tables 3 and 4).

The proportion of variance attributed to the variables included in the models was also estimated. The variance partition coefficient was defined as CPV=[(VA-VB)/VA]*100, where VA represents the variance of the null model and VB the variance of the model with more terms (21). The intraclass correlation index was defined as level 2 of variance (level 2 of variance + π^2/3). It provided an estimate of the total variance in outcomes that can be attributed to differences across states (Tables 3 and 4). 

The multilevel model added two levels of analysis: federative units as level 2 and individual characteristics as level 1. After the unadjusted model, in the raw analysis (Model 1), the individual variables that presented a p-value less than 0.20 were selected by the stepwise forward method for Model 2, with only those with a p-value lower than 0.05 being kept. Then, the following adjustments were made: model 2 + Municipal Human Development Index (Model 3), model 2 + Social Vulnerability Index (Model 4) and model 2 + Gini Index (Model 5). The multilevel analysis used the fixed effects model and random intersection to estimate the association. The Akaike information criterion was calculated as a measure of goodness of fit. All analyses were performed using Stata statistical software version 16.0.

## Results

Data from 4,336 Brazilian adolescents aged between 15 and 19 years were analyzed, of which 50.0% (95%CI 47.3; 52.8) were female. The age group of 15 to 17 years represented the majority of adolescents (59.7%; 95%CI 57.1; 62.3), with a higher proportion declaring themselves non-white (62.6%; 95%CI 59.7; 65.4), with completed elementary education (55.0%, 95%CI 52.3; 57.8), without a partner (97.7%; 95%CI 97.0; 98.3) and residing in urban areas (83.6%; 95%CI 81.5; 85.5). The majority of adolescents did not have a poor perception of their health (97.8%; 95%CI 96.9; 98.5) and did not have health insurance (81.9%; 95%CI 79.7; 83.9) (Table 1).

It is noteworthy that 25.6% (95%CI 23.3; 28.0) did not present any risk factor, while 23.6% (95%CI 21.4; 26.0) presented only the consumption of ultra-processed foods, configuring the isolated combination with the highest prevalence (Table 2).

Regarding simultaneity, the most prevalent combination for two risk behaviors included the consumption of ultra-processed foods and physical inactivity (13.3%; 95%CI 11.6; 15.3). For three simultaneous behaviors, the most prevalent was the combination of ultra-processed foods, physical inactivity, and habitual alcohol use (3.6%; 95%CI 2.7; 4.8). The combination of the four behaviors analyzed had a simultaneous prevalence of 0.7% (95%CI 0.4; 1.2) (Table 2).

The probability of having three or four simultaneous risk factors was lower for females (OR 0.44; 95%CI 0.32; 0.60) and in rural areas (OR 0.46; 95%CI 0.31; 0.70) and higher in older adolescents (OR 2.57; 95%CI 1.72; 3.83) and for adolescents without a partner (OR 3.83; 95%CI 1.10; 13.35). Furthermore, the chance of two factors occurring was higher among individuals with incomplete secondary education (OR 8.11; 95% CI 2.41; 27.26), followed by the group with incomplete primary education (OR 7.13; 95% CI 1.98; 25.64) (Table 3).

**Table 3:** Odds ratios (OR) and raw and adjusted 95% confidence intervals (95%CI) of concurrent health risk factors among adolescents by individual characteristics. Brasil, 2019 (n=4.336)

		Simultaneity of risk factors					
		Model 1^a^			Model 2^b^		
	Null model	1 Factor OR (95%CI)	2 Factors OR (95%CI)	3-4 Factors OR (95%CI)	1 Factor OR (95%CI)	2 Factors OR (95%CI)	3-4 Factors OR (95%CI)
**Fixed effect**							
**Individual characteristics**							
**Gender**							
Male		1.00	1.00	1.00	1.00	1.00	1.00
Female		0.85 (0.73; 0.88)	0.59 (0.46; 0.74)	0.42 (0.30; 0.57)	0.84 (0.72; 1.00)	0.59 (0.47; 0.75)	0.44 (0.32; 0.60)
**Age** (years)							
15-17		1.00	1.00	1.00	1.00	1.00	1.00
18-19		0.85 (0.81; 1.00)	1.03 (0.83; 1.27)	2.01 (1.44; 2.79)	0.96 (0.81; 1.14)	1.23 (0.98; 1.54)	2.57 (1.72; 3.83)
**Race/skin color**							
White		1.00	1.00	1.00	–	–	–
Non-white		1.22 (1.02; 1.46)	1.14 (0.88; 1.47)	0.81 (0.56; 1.18)	–	–	–
**Education**							
Illiterate		1.00	1.00	1.00	1.00	1.00	1.00
Incomplete elementary school		2.22 (1.13; 4.34)	6.54 (1.84; 23.28)	0.83 (0.25; 2.70)	2.23 (1.14; 4.36)	7.13 (1.98; 25.64)	1.10 (0.34; 3.55)
Incomplete high school		2.75 (1.38; 5.48)	7.56 (2.29; 24.87)	0.56 (0.18; 1.74)	2.79 (1.37; 5.65)	8.11 (2.41; 27.26)	0.67 (0.22; 2.04)
incomplete higher education		1.87 (0.97; 3.59)	5.61 (1.66; 18.90)	0.65 (0.21; 2.01)	1.96 (0.99; 3.89)	5.41 (1.53; 19.01)	0.48 (0.17; 1.37)
Completed higher education		2.20 (0.34; 13.94)	0.89 (0.05; 13.27)	1.40 (0.12; 15.93)	2.37 (0.37; 15.41)	0.90 (0.06; 14.69)	1.09 (0.11; 11.36)
**Marital status**							
With a partner		1.00	1.00	1.00	1.00	1.00	1.00
Without a partner		1.23 (0.72; 2.13)	1.88 (0.91; 3.91)	3.86 1.08; 13.80	1.11 (0.64; 1.91)	1.64 (0.80; 3.76)	3.83 (1.10; 13.35)
Urban							
Area		1.00	1.00	1.00	1.00	1.00	1.00
Rural		0.94 (0.78; 1.12)	0.72 (0.57; 0.92)	0.50 (0.32; 0.78)	0.94 (0.78; 1.12)	0.71 (0.56; 0.90)	0.46 (0.31; 0.70)
**Poor health perception**							
No		1.00	1.00	1.00	–	–	–
Yes		1.08 (0.55; 2.15)	1.37 (0.71; 2.65)	1.81 (0.58; 5.68)	–	–	–
**Health Insurance**							
Yes		1.00	1.00	1.00	–	–	–
No		1.17 (0.91; 1.51)	1.06 (0.81; 1.40)	1.05 (0.71; 1.55)	–	–	–
**Contextual characteristics**							
**Municipal Human Development Index**							
Mean		1.00	1.00	1.00			
High		1.41 (1.07; 1.85)	1.95 (1.63; 2.32)	2.01 (1.12; 3.62)			
Very high		2.51 (1.97; 3.20)	4.43 (3.57; 5.48)	7.28 (3.81; 13.92)			
**Social Vulnerability Index**							
Very low		1.00	1.00	1.00			
Low		0.79 (0.56; 1.11)	0.78 (0.50; 1.22)	0.47 (0.21; 1.04)			
Mean		0.62 (0.42; 0.91)	0.47 (0.30; 0.76)	0.25 (0.11; 0.58)			
**Gini Index**							
1st tertile		1.00	1.00	1.00			
2nd tertile		0.73 (0.49; 1.06)	0.54 (0.34; 0.87)	0.28 (0.15; 0.52)			
3rd tertile		0.72 (0.54; 0.97)	0.52 (0.37; 0.71)	0.35 (0.20; 0.62)			
**Random effect** (State)							
Variance (95% CI)	0.161 (0.099; 0.260)				0.153 (0.094; 0.248)		
Variance partition coefficient (%)					4.97		
Intraclass correlation coefficient	0.050				0.044		
Akaike information criterion	10,454.65				10,307.15		

^a^Model 1: bivariate analysis (raw); ^b^Model 2: analysis adjusted for individual characteristics.

The variance identified in the null model indicates that there is variation between Brazilian states regarding the probability of the risk behaviors assessed existing simultaneously among adolescents. The intraclass correlation index of 0.05 means that 5.0% of the total variance is due to the second level (states). After including individual variables (Model 2), the variance reduced from 0.16 to 0.15, suggesting that there are variables in the model that are unevenly distributed across states. These variables explained only 5.0% of the total variance (Table 3). 

In model 3, we noted that, when adjusted for individual characteristics, residing in states with a higher Human Development Index increases the chances of presenting three or four simultaneous risk factors (OR 7.28; 95%CI 3.81; 13.92). On the other hand, lower probabilities were observed for the mean values of the Social Vulnerability Index (OR 0.25; 95%CI 0.11; 0.58) and for the second tertile of the Gini Index (OR 0.28; 95%CI 0.15; 0.52) (Models 4 and 5) (Table 4). 

**Table 4:** Odds ratios (OR) and adjusted 95% confidence intervals (95%CI) of concurrent health risk factors among adolescents by socioeconomic indices. Brasil, 2019 (n=4.336)

	Simultaneity of risk factors									
	Model 3^a^ OR (95%CI)			Model 4^b^ OR (95%CI)				Model 5^c^ OR (95%CI)		
	1	2	3-4	1	2	3-4		1	2	3-4
**Fixed effect**										
**Individual characteristics**										
Gender										
Male	1.00	1.00	1.00	1.00	1.00	1.00		1.00	1.00	1.00
Female	0.84 (0.72; 1.00)	0.59 (0.47; 0.75)	0.45 (0.33; 0.63)	0.84 (0.72; 0.99)	0.59 (0.46; 0.74)	0.44 (0.32; 0.60)		0.84 (0.71; 0.99)	0.58 (0.46; 0.74)	0.43 (0.31; 0.59)
**Age** (years)										
15-17	1.00	1.00	1.00	1.00	1.00	1.00		1.00	1.00	1.00
18-19	0.96 (0.81; 1.14)	1.24 (1.00; 1.56)	2.72 (1.80; 4.13)	0.96 (0.81; 1.14)	1.24 (0.99; 1.55)	2.66 (1.76; 4.01)		0.96 (0.81; 1.14)	1.25 (1.00; 1.55)	2.64 (1.74; 4.00)
**Education**										
Illiterate	1.00	1.00	1.00	1.00	1.00	1.00		1.00	1.00	1.00
Incomplete elementary school	2.24 (1.16; 4.33)	7.22 (2.01; 25.10)	1.09 (0.33; 3.58)	2.23 (1.15; 4.35)	7.30 (2.01; 26.53)	1.07 (0.33; 3.42)		2.24 (1.15; 4.36)	7.37 (2.00; 27.1)	1.28 (0.35; 4.63)
Incomplete high school	2.81 (1.40; 5.65)	8.11 (2.41; 27.26)	0.62 (0.20; 1.91)	2.80 (1.39; 5.65)	8.29 (2.45; 28.09)	0.63 (0.21; 1.93)		2.81 (1.39; 5.69)	8.25 (2.40; 28.32)	0.74 (0.22; 2.47)
incomplete higher education	2.00 (1.02; 3.91)	5.31 (1.50; 18.72)	0.41 (0.14; 1.19)	1.98 (1.00; 3.92)	5.53 (1.55; 19.68)	0.43 (0.16; 1.19)		2.44 (0.38; 15.7)	5.45 (1.52; 19.61)	0.51 (0.16; 1.62)
Completed higher education	2.41 (0.39; 15.13)	0.84 (0.05; 13.76)	0.80 (0.09; 7.00)	2.42 (0.37; 15.83)	0.93 (0.06; 15.02)	0.88 (0.11; 6.92)		0.91 (0.76; 1.1)	0.91 (0.06; 15.0)	1.10 (0.11; 11.00)
**Marital status**										
With a partner	1.00	1.00	1.00	1.00	1.00	1.00		1.00	1.00	1.00
Without a partner	1.11 (0.65; 1.91)	1.60 (0.78; 3.26)	3.77 (1.06; 13.37)	0.92 (0.77; 1.11)	0.72 (0.57; 0.92)	0.49 (0.32; 0.75)		1.12 (0.65; 1.93)	0.74 (0.59; 0.93)	0.54 (0.35; 0.82)
**Urban**										
Area	1.00	1.00	1.00	1.00	1.00	1.00		1.00	1.00	1.00
Rural	0.91 (0.76; 1.10)	0.74 (0.58; 0.93)	0.52 (0.34; 0.81)	1.12 (0.65; 1.92)	1.65 (0.81; 3.36)	3.87 (1.10; 13.55)		0.72 (0.50; 1.05)	1.68 (0.83; 3.41)	3.86 (1.09; 13.69)
**Contextual Features**										
**Municipal Human Development Index**										
Mean	1.00	1.00	1.00							
High	1.39 (1.10; 1.76)	1.90 (1.63; 2.22)	1.87 (1.03; 3.40)							
Very high	2.45 (2.00; 3.01)	4.18 (3.36; 5.20)	6.83 (3.39; 13.75)							
**Social Vulnerability Index**										
Very low				1.00	1.00	1.00				
Low				0.80 (0.57; 1.11)	0.79 (0.52; 1.21)	0.45 (0.22; 0.96)				
Mean				0.62 (0.43; 0.90)	0.48 (0.31; 0.75)	0.25 (0.12; 0.54)				
**Gini Index**										
1st tertile							1.00		1.00	1.00
2nd tertile								0.73 (0.54; 0.98)	0.54 (0.35; 0.83)	0.28 (0.15; 0.51)
3rd tertile								0.84 (0.71; 0.99)	0.52 (0.38; 0.70)	0.34 (0.19; 0.60)
**Random effect** (State)										
Variance (95% CI)	0.04 (0.01; 0.13)			0.11 (0.07; 0.18)				0.10 (0.05; 0.18)		
Variance partition coefficient (%)	75.16			31.68				39.75		
Intraclass correlation coefficient	0.012			0.032				0.029		
Akaike information criterion	10,249.91			10,276.1				10,253.56		

^a^Model 3: model 2 adjusted by the Municipal Human Development Index; ^b^Model 4: model 2 adjusted by the Social Vulnerability Index; ^c^Model 5: model 2 adjusted by the Gini Index.

With the introduction of contextual variables, the variance decreased from 0.16 to 0.04 (Model 3), 0.11 (Model 4) and 0.10 (Model 5). The greatest reduction was due to the adjustment of model 3 (CPV 75.2%), as well as the lowest intraclass correlation index (0.012) and lowest Akaike information criterion (10,249.91), indicating that this was the best model to explain the variability existing between Brazilian states for the simultaneous prevalence of risk factors.

## Discussion

The prevalence of simultaneous health risk behaviors among adolescents was influenced by individual characteristics (male sex, living in an urban area, older age, not having a partner and having a higher level of education) and contextual characteristics. It was observed that states with a higher Human Development Index (HDI) have a higher prevalence of multiple risk behaviors, while greater social vulnerability and economic inequality are associated with lower chances of simultaneity. The variation between Brazilian states was partially explained by contextual factors, with the HDI demonstrating greater influence, which highlights the importance of regional inequalities in determining these behaviors.

Despite presenting limitations inherent to its cross-sectional design, the use of secondary and self-reported data (information bias and underestimation of prevalence), this study offers relevant contributions to the understanding of risk behaviors among Brazilian adolescents. The broad representativeness of the sample and the multilevel analysis highlight the influence of contextual factors and provide important support for public policies aimed at reducing these behaviors.

These findings are consistent with studies conducted in other contexts (4,5), which indicated similar patterns of simultaneity of risk behaviors, particularly among male and non-white adolescents. A study conducted in 140 countries (5) identified a high prevalence of individual risk behaviors, such as insufficient consumption of fruits and vegetables and physical inactivity. Another study, analyzing 89 countries, revealed that the simultaneity of lifestyle risk factors, including smoking and alcohol consumption, was predominant among male adolescents, with significant regional differences, but high prevalence in different regions of the globe (22). This global phenomenon suggests that the simultaneity of risk factors is a cross-cutting issue, although with different manifestations according to socioeconomic and cultural conditions.

Among the most prevalent isolated risk behaviors in this study, the high consumption of ultra-processed foods stands out, a dietary pattern also frequently observed in studies conducted with Brazilian adolescents (23-25). In the general population, consumption of these foods tends to decrease with age and increase with education and income (26). Among adolescents, this habit is strongly associated with excessive screen time, childhood eating habits and type of school, being more prevalent among students in private schools (24).

The most frequent combination of risk behaviors observed — consumption of ultra-processed foods and physical inactivity — reiterates the findings of other research in the country (27) and reflects a global pattern identified elsewhere, where low consumption of fruits and vegetables is commonly combined with physical inactivity (5). It is worth noting that although the lack of physical activity is a problem, there is evidence that sedentary habits themselves are even more harmful and are more strongly associated with the consumption of ultra-processed foods (25). 

The simultaneity of three risk behaviors (consumption of ultra-processed foods, physical inactivity, and habitual alcohol use) found corroborates findings in the Brazilian context (28) and in global studies (22), where alcohol consumption stands out as a relevant component in the combinations of risk factors. Although the simultaneity of risk behaviors in adolescents presents a low prevalence, decreasing with the increase of associated factors, its manifestation varies according to individual and contextual conditions.

In rural areas there is a lower chance of accumulation of risk factors, which can be attributed to the uncomplicated way of life in the countryside (25). However, despite a more natural diet and an environment less prone to physical inactivity, there has been an increase in alcoholism and smoking, associated with a poor perception of risk, which results from the limited supply of information. In contrast, urban areas present a different scenario, in which greater exposure to ultra-processed foods, sedentary lifestyle and the accelerated pace of life favor the adoption of harmful habits and restrict the options for healthy choices (29).

Advanced age among adolescents is associated with greater simultaneity of risk factors for chronic diseases, possibly due to greater autonomy and consequent greater vulnerability, with the adoption of less healthy habits (10, 30). This relationship is intensified among adolescents with higher levels of education, who are more likely to accumulate risky behaviors, possibly due to increased social and behavioral freedom (31,32). However, it is important to highlight that the effect of education on risk behavior was moderate when adjusted for the HDI. This adjustment suggests that the level of socioeconomic development may mitigate the negative effects associated with increasing age and education.

Adolescents without a partner, living in states with a high HDI, were more likely to accumulate risk factors. Although the relationship between unhealthy lifestyle habits and marital status among adolescents has not yet been widely explored in the literature, the association between being single and greater exposure to risk behaviors is well established, especially in the age group analyzed in this study (33). 

There is evidence that socioeconomic development, with improvements in education, health, and infrastructure, is associated with more effective control of chronic diseases, due to the greater adoption of healthy behaviors by the population, resulting in lower mortality rates (34). However, the results of this study indicate that in states with greater development, the chances of simultaneous presentation of risk factors among adolescents increase, with a reverse effect when analyzed from the perspective of social vulnerability, since adolescents who lived in regions with higher Social Vulnerability and Gini Indexes had a lower probability of occurrence of isolated and similar risk behaviors. 

It is worth highlighting that the results of this study converge with the findings of the 2015 Brazilian National School Health Survey (27), which identified that the accumulation of risk factors among adolescents was greater among residents in cities of more developed urban areas of the country. This suggests that risk behaviors in this population tend to be accentuated in areas of greater socioeconomic development, in which capital accumulation is often associated with behaviors that are harmful to health (31). Countries in Latin America and the Caribbean with higher Gross Domestic Product and HDI, for example, have a higher prevalence of overweight and obesity among adolescents (35). Likewise, more developed areas are related to a higher prevalence of high blood pressure, a condition largely influenced by habits such as high consumption of ultra-processed foods, a sedentary lifestyle and alcohol and tobacco consumption (36,37).

 Although social vulnerability is generally associated with worse health indicators, due to the difficulty in accessing essential resources (38), the relationship with the accumulation of risk factors among adolescents presents a specific dynamic, and the results of this study suggest that less developed regions can act as protective factors against the accumulation of these behaviors. This effect needs to be further explored, but it can be explained by several social and cultural mechanisms present in these contexts, such as community cohesion, family support and cultural values that tend to mitigate the prevalence of risk behaviors (39).

Finally, we highlight the influence of individual variables on the concurrence of risk factors present in Brazilian adolescents, suggesting that the chances of these adolescents accumulating risk factors vary when adjusted for contextual variables and increase with the greater development of the region in which they live. These results reinforce the need for comprehensive health promotion and social well-being policies capable of addressing multiple risk factors for chronic non-communicable diseases among adolescents in different regions of the country. Future studies can explore the complexity of multilevel factors affecting this group, including individual, family, socioeconomic, and market levels.

## Data Availability

The database and the codes used for the analysis in the research are available at https://www.ibge.gov.br/estatisticas/sociais/saude/29540-2013-pesquisa-nacional-de-saude.html?edicao=9177&t=microdados.
