# Supplementary material for: Individual and contextual characteristics associated with concurrent risk factors among adolescents: a multilevel cross-sectional study based on the 2019 Brazilian National Health Survey
Source: Epidemiol Serv Saude. 2025 Sep 8;34:e20240453. doi: 10.1590/S2237-96222025v34e20240453.en (PMC12435959; doi:10.1590/S2237-96222025v34e20240453.en)
Supplement: Translation

# Translation: Características individuais e contextuais associadas a fatores de risco simultâneos entre adolescentes: estudo transversal multinível baseado na Pesquisa Nacional de Saúde 2019

**DOI:** 10.1590/S2237-96222025v34e20240453.pt

**Authors:** Ruàn Éverton de Souza Silva, Eduardo Araujo Lima, Antonio Valdeir Lopes da Silva, Shelda Santos Silva, Jéssica Fernanda de Sousa, Edina Araújo Rodrigues Oliveira, Danilla Michelle Costa e Silva, Mailson Fontes de Carvalho, Rumão Batista Nunes de Carvalho

**Affiliations:** 1 Universidade Federal do Piauí, Picos, PI, Brasil; 2 Universidade Federal do Recôncavo da Bahia, Salvador, BA, Brasil

**Keywords:** Fatores de Risco, Fatores Socioeconômicos, Desigualdades de Saúde, Adolescente, Estudos Transversais

## Abstract

**Objetivo:**

Avaliar a simultaneidade de comportamentos de risco para doenças crônicas não transmissíveis e sua associação com características individuais e contextuais em adolescentes brasileiros.

**Métodos:**

Estudo transversal, com dados da Pesquisa Nacional de Saúde, 2019. Analisou-se a simultaneidade dos fatores de consumo de alimentos ultraprocessados, nível de atividade física, tabagismo e uso de álcool, segundo características individuais e contextuais, estimando-se as razões de chances (*odds ratio* - OR) e respectivos intervalos de confiança de 95% (IC95%) para os efeitos fixos e variância e IC95% para os efeitos aleatórios, através da regressão logística politômica multinível.

**Resultados:**

Entre os 4.336 adolescentes avaliados, a combinação mais prevalente de dois comportamentos de risco incluiu o consumo de alimentos ultraprocessados e o nível insuficiente de atividade física (13,3%; IC95% 11,6; 15,3). A combinação mais frequente de três fatores incluiu esses comportamentos somados ao uso habitual de álcool (3,6%; IC95% 2,7; 4,8). As chances das combinações de três ou quatro fatores foram menores no sexo feminino (OR 0,44; IC95% 0,32; 0,60) e em áreas rurais (OR 0,46; IC95% 0,31; 0,70) e maiores em adolescentes de maior idade (OR 2,57; IC95% 1,72; 3,83), maior escolaridade (OR 8,11; IC95% 2,41; 27,26) e que vivem sem companheiros (OR 3,83; IC95% 1,10; 13,35). Índices de Desenvolvimento Humano elevados aumentaram essas chances (OR 7,28; IC95% 3,81; 13,92), enquanto altos Índices de Vulnerabilidade Social (OR 0,25; IC95% 0,11; 0,58) e de Gini (OR 0,28; IC95% 0,15; 0,52) reduziram.

**Conclusão:**

A ocorrência de múltiplos comportamentos de risco em adolescentes é mais provável entre os mais velhos, do sexo masculino, solteiros e com maior escolaridade, especialmente em áreas de maior desenvolvimento socioeconômico.

**Aspectos éticos**

Esta pesquisa utilizou bancos de dados de domínio público e anonimizados.:

## Introdução

As doenças crônicas não transmissíveis representam um importante problema de saúde pública em todo o mundo e se mantêm associadas a impactos expressivos na economia das famílias e da sociedade (1). No Brasil, o impacto dessas doenças evidencia uma crise crescente no sistema de saúde, sendo responsáveis por quase 70,0% da mortalidade precoce no país (2).

A adolescência, etapa de transição entre a infância e a vida adulta, é marcada por intensas mudanças físicas, emocionais e sociais que desempenham papel central na adoção de comportamentos de risco, como má alimentação, tabagismo, sedentarismo e consumo de álcool (3). Essa fase é caracterizada pela vulnerabilidade psicológica, busca de aceitação e autonomia, tornando os jovens mais suscetíveis à experimentação de práticas prejudiciais à saúde (3,4).

Entre jovens de 10 a 20 anos, a inatividade física e o comportamento sedentário, sobretudo decorrente da elevada exposição às telas, estão ligados ao aumento da obesidade, baixo consumo de frutas e vegetais, consumo excessivo de sal e bebidas açucaradas, depressão e redução da qualidade de vida (4,5). Esses comportamentos, quando fixados nessa fase, estão relacionados a cerca de dois terços das mortes prematuras em adultos (5).

Características individuais como sexo, idade, raça/cor da pele e educação materna apresentam-se fortemente associadas aos comportamentos de risco (6) e reforçam a importância da vigilância desses fatores, visando não apenas o acompanhamento de grupos prioritários, mas também a promoção da saúde de indivíduos expostos (7). Aspectos contextuais também podem influenciar diretamente os comportamentos de risco para doenças crônicas não transmissíveis. Programas de promoção da saúde de qualidade (8), frequência escolar e o apoio dos pais (9) ajudam a reduzir esses comportamentos de risco. No revés, iniquidades como baixos níveis de escolaridade, dificuldades de acesso ao mercado de trabalho e a má distribuição de renda contribuem para a prevalência dessas doenças (10).

Apesar do impacto significativo de fatores individuais e contextuais na adoção de comportamentos de risco para doenças crônicas não transmissíveis, ainda são poucas as pesquisas que analisam a manifestação simultânea desses fatores, considerando os contextos associados (8,9). Este estudo tem objetivo de avaliar a simultaneidade de comportamentos de risco para doenças crônicas não transmissíveis e associar com características individuais e contextuais em adolescentes brasileiros.

## Métodos

### Delineamento 

Trata-se de um estudo transversal de base populacional, utilizando dados da Pesquisa Nacional de Saúde (PNS) de 2019. 

### Contexto

A PNS 2019, realizada pelo Instituto Brasileiro de Geografia e Estatística (IBGE) em parceria com o Ministério da Saúde, é um inquérito representativo da população brasileira, que coletou dados sobre determinantes e necessidades de saúde de brasileiros com 15 anos ou mais, residentes em domicílios permanentes, usando amostragem por conglomerado em três estágios: estratificação de unidades primárias de amostragem (setores censitários com probabilidade proporcional ao número de domicílios), seleção aleatória de domicílios e, por fim, seleção aleatória de um morador (11).

### Participantes 

Neste estudo, foram utilizados dados de adolescentes de 15 a 19 anos (12), residentes em domicílios particulares permanentes em todas as Unidades Federativas do país. 

### Variáveis 

Utilizou-se variáveis individuais e contextuais para analisar o desfecho, que foi a simultaneidade de fatores de risco à saúde. Os comportamentos de risco individuais considerados incluíram o consumo de alimentos ultraprocessados, o nível insuficiente de atividade física no lazer, o tabagismo e o uso habitual de álcool. Foram avaliadas também as características individuais como: sexo (masculino; feminino), idade em anos (15 a 17; 18 a 19), raça/cor da pele (branca; não branca), escolaridade (sem escolaridade; fundamental incompleto; fundamental completo; médio completo; superior completo), estado civil (com companheiro; sem companheiro), zona de residência (urbana; rural), percepção de saúde ruim (não; sim) e cobertura de plano de saúde (não; sim).

Quanto às características contextuais, foram utilizados os indicadores de equidade: Índice de Desenvolvimento Humano Municipal (médio; alto; muito alto) Índice de Vulnerabilidade Social (muito baixo; baixo; médio) e o Índice de Gini (1º tercil; 2º tercil; 3º tercil). 

### Fontes de dados e mensuração 

Os dados para análise das variáveis individuais provêm da PNS 2019, de acesso aberto através do sítio do IBGE. Este inquérito nacional realizou coleta dos dados domiciliar, realizada por entrevistadores treinados que aplicaram questionário contendo três instrumentos: a) domiciliar, b) de todos os moradores do domicílio e c) individual. 

O consumo de alimentos ultraprocessados foi caracterizado pela ingestão frequente, definida como cinco ou mais vezes por semana, de produtos como biscoitos/bolachas recheadas, chocolates, gelatinas, balas e similares, refrigerantes/sucos artificiais e lanches que substituem o almoço como sanduíches, salgados, pizzas, cachorro-quente, entre outros (0 = não; 1 = sim). O nível insuficiente de atividade física no lazer foi considerado quando relatado não realizar, pelo menos, 300 minutos de atividades físicas semanalmente, considerando somente atividades realizadas no tempo livre (0 = não; 1 = sim) (13). Quanto ao tabagismo e uso habitual de álcool, considerou-se como fator de risco qualquer consumo desses indicadores nos últimos 30 dias anteriores à pesquisa (0 = não; 1 = sim), tendo em vista a maior vulnerabilidade dos adolescentes aos danos causados por essas substâncias, cuja venda é proibida para essa faixa etária no Brasil (14).

A percepção de saúde ruim foi obtida pela classificação da própria saúde em “muito boa”, “boa”, “regular”, “ruim” ou “muito ruim”. Nesta análise, separou-se essa classificação em duas categorias: saúde boa (regular, boa e muito boa) e saúde ruim (ruim e muito ruim) (0 = não; 1 = sim). A cobertura de plano de saúde foi avaliada quanto a afirmativa sobre disponibilidade de plano de saúde particular, de empresa ou órgão público (0 = sim; 1 = não). 

No tocante aos aspectos contextuais, os dados foram obtidos do Atlas do Desenvolvimento Humano no Brasil e do Instituto de Pesquisa Econômica Aplicada. O Índice de Vulnerabilidade Social é calculado a partir de dezesseis indicadores agrupados em três subíndices: infraestrutura urbana, capital humano e renda e trabalho, variando entre 0,000 e 1,000, sendo que o 0 indica ausência de vulnerabilidade social e o 1 representa o nível máximo de vulnerabilidade (0 a 0,200 - vulnerabilidade social muito baixa; 0,201 e 0,300 - vulnerabilidade social baixa; 0,301 e 0,400 - vulnerabilidade social média; 0,401 e 0,500 - vulnerabilidade social alta, e >0,500 - vulnerabilidade social muito alta) (15).

O Índice de Desenvolvimento Humano Municipal também varia de 0,000 a 1,000, em que, quanto mais próximo de 1, maior é o desenvolvimento humano de determinada região geográfica. É classificado em muito baixo (0,000-0,499), baixo (0,500-0,599), médio (0,600-0,699), alto (0,700-0,799) e muito alto (0,800-1,000) (16).

O Índice de Gini é usado como parâmetro para mensurar a desigualdade de renda entre os indivíduos de uma população, sendo quantificado entre 0 e 1, em que, valores mais próximos de 0 indicam menor desigualdade de renda, e valores mais próximos de 1 indicam maior desigualdade (17).

Neste estudo não foram observados valores para as classificações baixo e muito baixo para o Índice de Desenvolvimento Humano Municipal e, alto e muito alto para o Índice de Vulnerabilidade Social. 

### Tamanho do estudo

O tamanho amostral da PNS 2019 considerou a precisão desejada para estimar os principais fatores de risco e proteção para doenças crônicas não transmissíveis, prevalência da doença, acesso aos serviços de saúde, entre outras variáveis. A amostra esperada foi de 108.525 domicílios, e os dados foram coletados em 94.114, com uma taxa de não resposta de 6,4% (18). Foram entrevistados 90.846 indivíduos (11), dos quais 4.336 adolescentes entre 15 e 19 anos, que compõem esta análise. 

### Métodos estatísticos

Inicialmente, realizou-se uma análise descritiva com frequências simples e relativas das características sociodemográficas dos adolescentes (Tabela 1). Ademais, analisou-se a ocorrência exclusiva de todas as possíveis combinações entre os quatro fatores de risco, incluindo a ausência de fatores e seus respectivos intervalos de confiança (Tabela 2).

**Tabela 2:** Proporções e intervalos de confiança de 95% (IC95%) das combinações de comportamentos de risco à saúde entre adolescentes. Brasil, 2019 (n=4.336)

Fatores de Risco	Alimentos Ultraprocessados	Tabagismo	Inatividade física	Uso habitual de álcool	Prevalência (IC95%)
0	-	-	-	-	25,6 (23,3; 28,0)
1	-	-	-	+	4,6 (3,5; 6,0)
1	-	-	+	-	15,4 (13,6; 17,4)
1	-	+	-	-	0,6 (0,3; 0,9)
1	+	-	-	-	23,6 (21,4; 26,0)
2	-	-	+	+	3,1 (2,4; 4,1)
2	-	+	-	+	1,2 (0,7; 2,1)
2	-	+	+	-	0,2 (0,1; 0,7)
2	+	-	-	+	4,6 (3,3; 6,3)
2	+	-	+	-	13,3 (11,6; 15,3)
2	+	+	-	-	1,0 (0,6; 1,5)
3	-	+	+	+	0,4 (0,2; 0,8)
3	+	-	+	+	3,6 (2,7; 4,8)
3	+	+	-	+	1,8 (1,0;3,2)
3	+	+	+	-	0,3 (0,2;0,5)
4	+	+	+	+	0,7 (0,4;1,2)

**Tabela 1:** Proporções e intervalos de confiança de 95% (IC95%) de adolescentes segundo características sociodemográficas. Brasil, 2019 (n=4.336)

Variáveis	Prevalência (%)	(IC95%)
Sexo		
Masculino	50,0	(47,2; 52,7)
Feminino	50,0	(47,3; 52,8)
**Idade** (anos)		
15-17	59,7	(57,1; 62,3)
18-19	40,3	(37,7; 42,9)
**Raça/cor da pele**		
Branca	37,4	(34,6; 40,3)
Não branca	62,6	(59,7; 65,4)
Escolaridade		
Sem escolaridade	1,3	(0,7; 2,5)
Fundamental incompleto	18,7	(16,7; 21,0)
Fundamental completo	55,0	(52,3; 57,8)
Médio completo	24,7	(22,5; 27,2)
Superior completo	0,1	(0,0; 0,5)
**Estado civil**		
Com companheiro	2,3	(1,7; 3,0)
Sem companheiro	97,7	(97,0; 98,3)
Zona		
Urbana	83,6	(81,5; 85,5)
Rural	16,4	(14,5; 18,5)
**Percepção de saúde ruim**		
Não	97,8	(96,9; 98,5)
Sim	2,2	(1,5; 3,1)
Plano		
Sim	18,1	(16,1; 20,3)
Não	81,9	(79,7; 83,9)

Em amostras complexas, os pesos de amostragem refletem a probabilidade de seleção nos conglomerados em amostras hierárquicas, diferindo-os dos de frequência (19). O não escalonamento desses pesos pode enviesar as estimativas. Assim, optou-se por ajustá-los apenas no primeiro nível, já que no segundo nível não afeta significativamente as estimativas dos parâmetros e seus erros padrão (20). 

Como a amostra estudada apresenta estratos de tamanho pequeno sem o plano amostral, aplicou-se a fórmula de escalonamento dos pesos, compreendida por wij*=wij(nj/∑wij) (19,20), donde wij* representa o peso escalonado para o indivíduo i no estado j; wij simboliza o peso não escalonado para o indivíduo i no estado j; e nj é o número de unidades amostrais no estado j. A variável de estrato foi utilizada para indicar a estrutura de agrupamento, enquanto a variável de peso do domicílio com correção de não entrevista sem calibração pela projeção de população, constituiu o peso de amostragem individual (19,20).

A estimativa da prevalência e respectivo intervalo de confiança de 95% da simultaneidade de comportamentos de risco e de cada fator de risco foram realizados de forma independente. A regressão logística politômica multinível também foi executada, utilizando o modelo logit multinomial (mlogit) pela aplicação da modelagem de equações estruturais generalizadas (gsem) para investigar a associação entre a simultaneidade de fatores de risco (1, 2, 3/4 fatores de risco) e as variáveis individuais e contextuais. Estimaram-se as razões de chances (odds ratio - OR) e respectivos intervalos de confiança de 95% (IC95%) para os efeitos fixos e variância e IC95% para os efeitos aleatórios (Tabelas 3 e 4).

Também foi estimada a proporção da variância atribuída às variáveis incluídas nos modelos. O coeficiente de partição de variância foi definido como CPV=[(VA-VB)/VA]*100, onde VA representa a variância do modelo nulo e VB a variância do modelo com mais termos (21). Já o índice de correlação intraclasse foi definido como nível 2 de variância (nível 2 de variância + π^2/3). Ele forneceu uma estimativa de variância total nos desfechos que pode ser atribuída a diferenças entre os estados (Tabelas 3 e 4). 

O modelo multinível acrescentou dois níveis de análise: unidades federativas como nível 2 e características individuais como nível 1. Após o modelo não ajustado, na análise bruta (Modelo 1), as variáveis individuais que apresentaram um p-valor menor que 0,20 foram selecionadas pelo método stepwise forward para o Modelo 2, mantidas apenas aquelas com p-valor inferior a 0,05. Em seguida, fizeram-se os seguintes ajustes: modelo 2 + Índice de Desenvolvimento Humano Municipal (Modelo 3), modelo 2 + Índice de Vulnerabilidade Social (Modelo 4) e modelo 2 + Índice de Gini (Modelo 5). A análise multinível utilizou o modelo de efeitos fixos e interseção aleatória para estimar a associação. Calculou-se o critério de informação de Akaike como uma medida de qualidade de ajuste. Todas as análises foram realizadas no software estatístico Stata versão 16.0.

## Resultados

Foram analisados dados de 4.336 adolescentes brasileiros, entre 15 e 19 anos dos quais 50,0% (IC95% 47,3; 52,^8^) era do sexo feminino. A faixa etária de 15 a 17 anos representava a maioria dos adolescentes (59,7%; IC95% 57,1; 62,^3^), maior proporção autodeclarando-se não brancos (62,6%; IC95% 59,7; 65,^4^), com ensino fundamental completo (55,0%; IC95% 52,3; 57,^8^), sem companheiro (97,7%; IC95% 97,0; 98,^3^) e residindo em áreas urbanas (83,6%; IC95% 81,5; 85,^5^). A maior parcela dos adolescentes não tinha percepção ruim da saúde (97,8%; IC95% 96,9; 98,^5^) e não possuía plano de saúde (81,9%; IC95% 79,7; 83,^9^) (Tabela 1).

Destaca-se que 25,6% (IC95% 23,3; 28,0) não apresentavam qualquer fator de risco, ao passo que 23,6% (IC95% 21,4; 26,0) apresentavam unicamente o consumo de alimentos ultraprocessados, configurando a combinação isolada de maior prevalência (Tabela 2).

Quanto à simultaneidade, a combinação mais prevalente para dois comportamentos de risco incluiu o consumo de alimentos ultraprocessados e a inatividade física (13,3%; IC95% 11,6; 15,^3^). Para três comportamentos simultâneos, a mais prevalente foi a combinação de alimentos ultraprocessados, inatividade física e uso habitual de álcool (3,6%; IC95% 2,7; 4,^8^). Já a combinação dos quatro comportamentos analisados teve prevalência simultânea de 0,7% (IC95% 0,4; 1,^2^) (Tabela 2).

As probabilidades de existência de três ou quatro fatores de risco simultâneos foram menores no sexo feminino (OR 0,44; IC95% 0,32; 0,60) e em áreas rurais (OR 0,46; IC95% 0,31; 0,70) e maiores nos adolescentes mais velhos (OR 2,57; IC95% 1,72; 3,83) e sem companheiros (OR 3,83; IC95% 1,10; 13,^35^). Além disso, a chance de ocorrência de dois fatores foi maior entre indivíduos com ensino médio incompleto (OR 8,11; IC95% 2,41; 27,^26^), seguido pelo grupo com ensino fundamental incompleto (OR 7,13; IC95% 1,98; 25,64) (Tabela 3).

**Tabela 3:** Razões de chances (*Odds ratio*, OR) e intervalos de confiança de 95% (IC95%) brutas e ajustadas de fatores de risco à saúde simultâneos entre adolescentes pelas características individuais. Brasil, 2019 (n=4.336)

		Simultaneidade dos fatores de risco					
		Modelo 1^a^			Modelo 2^b^		
	Modelo nulo	1 Fator OR (IC95%)	2 Fatores OR (IC95%)	3-4 Fatores OR (IC95%)	1 Fator OR (IC95%)	2 Fatores OR (IC95%)	3-4 Fatores OR (IC95%)
**Efeito fixo**							
**Características Individuais**							
**Sexo**							
Masculino		1,00	1,00	1,00	1,00	1,00	1,00
Feminino		0,85 (0,73; 0,88)	0,59 (0,46; 0,74)	0,42 (0,30; 0,57)	0,84 (0,72; 1,00)	0,59 (0,47; 0,75)	0,44 (0,32; 0,60)
**Idade** (anos)							
15-17		1,00	1,00	1,00	1,00	1,00	1,00
18-19		0,85 (0,81; 1,00)	1,03 (0,83; 1,27)	2,01 (1,44; 2,79)	0,96 (0,81; 1,14)	1,23 (0,98; 1,54)	2,57 (1,72; 3,83)
**Raça/cor da pele**							
Branca		1,00	1,00	1,00	–	–	–
Não branca		1,22 (1,02; 1,46)	1,14 (0,88; 1,47)	0,81 (0,56; 1,18)	–	–	–
**Escolaridade**							
Analfabeto		1,00	1,00	1,00	1,00	1,00	1,00
Fundamental incompleto		2,22 (1,13; 4,34)	6,54 (1,84; 23,28)	0,83 (0,25; 2,70)	2,23 (1,14; 4,36)	7,13 (1,98; 25,64)	1,10 (0,34; 3,55)
Médio incompleto		2,75 (1,38; 5,48)	7,56 (2,29; 24,87)	0,56 (0,18; 1,74)	2,79 (1,37; 5,65)	8,11 (2,41; 27,26)	0,67 (0,22; 2,04)
Superior incompleto		1,87 (0,97; 3,59)	5,61 (1,66; 18,90)	0,65 (0,21; 2,01)	1,96 (0,99; 3,89)	5,41 (1,53; 19,01)	0,48 (0,17; 1,37)
Superior completo		2,20 (0,34; 13,94)	0,89 (0,05; 13,27)	1,40 (0,12; 15,93)	2,37 (0,37; 15,41)	0,90 (0,06; 14,69)	1,09 (0,11; 11,36)
**Estado civil**							
Com companheiro		1,00	1,00	1,00	1,00	1,00	1,00
Sem companheiro		1,23 (0,72; 2,13)	1,88 (0,91; 3,91)	3,86 1,08; 13,80	1,11 (0,64; 1,91)	1,64 (0,80; 3,76)	3,83 (1,10; 13,35)
Zona							
Urbana		1,00	1,00	1,00	1,00	1,00	1,00
Rural		0,94 (0,78; 1,12)	0,72 (0,57; 0,92)	0,50 (0,32; 0,78)	0,94 (0,78; 1,12)	0,71 (0,56; 0,90)	0,46 (0,31; 0,70)
**Percepção de saúde ruim**							
Não		1,00	1,00	1,00	–	–	–
Sim		1,08 (0,55; 2,15)	1,37 (0,71; 2,65)	1,81 (0,58; 5,68)	–	–	–
**Plano de Saúde**							
Sim		1,00	1,00	1,00	–	–	–
Não		1,17 (0,91; 1,51)	1,06 (0,81; 1,40)	1,05 (0,71; 1,55)	–	–	–
**Característica contextuais**							
**Índice de Desenvolvimento Humano Municipal**							
Médio		1,00	1,00	1,00			
Alto		1,41 (1,07; 1,85)	1,95 (1,63; 2,32)	2,01 (1,12; 3,62)			
Muito alto		2,51 (1,97; 3,20)	4,43 (3,57; 5,48)	7,28 (3,81; 13,92)			
**Índice de Vulnerabilidade Social**							
Muito baixo		1,00	1,00	1,00			
Baixo		0,79 (0,56; 1,11)	0,78 (0,50; 1,22)	0,47 (0,21; 1,04)			
Médio		0,62 (0,42; 0,91)	0,47 (0,30; 0,76)	0,25 (0,11 ;0,58)			
**Índice de Gini**							
1° tercil		1,00	1,00	1,00			
2º tercil		0,73 (0,49; 1,06)	0,54 (0,34; 0,87)	0,28 (0,15; 0,52)			
3º tercil		0,72 (0,54; 0,97)	0,52 (0,37; 0,71)	0,35 (0,20; 0,62)			
**Efeito aleatório** (Estado)							
Variância (IC95%)	0,161 (0,099; 0,260)				0,153 (0,094; 0,248)		
Coeficiente de partição de variância (%)					4,97		
Coeficiente de correlação intraclasse	0,050				0,044		
Critério de informação de Akaike	10.454,65				10.307,15		

^a^ Modelo 1: análise bivariada (bruta); ^b^Modelo 2: análise ajustada pelas características individuais.

A variância identificada no modelo nulo indica que há uma variação entre os estados brasileiros quanto à probabilidade de existir, simultaneamente, os comportamentos de risco avaliados entre os adolescentes. O índice de correlação intraclasse de 0,05 significa que 5,0% da variância total é devida ao segundo nível (estados). Após a inclusão das variáveis individuais (Modelo 2), a variância reduziu de 0,16 para 0,^15^, sugerindo que há variáveis no modelo desigualmente distribuídas entre os estados. Essas variáveis explicaram apenas 5,0% da variância total (Tabela 3). 

No modelo 3, observa-se que, quando ajustado pelas características individuais, residir em estados com maior Índice de Desenvolvimento Humano aumenta as chances de apresentarem três ou quatro fatores de risco simultâneos (OR 7,28; IC95% 3,81; 13,92). Por outro lado, menores probabilidades foram observadas para os valores médios do Índice de Vulnerabilidade Social (OR 0,25; IC95% 0,11; 0,58) e para o segundo tercil do Índice de Gini (OR 0,28; IC95% 0,15; 0,52) (Modelos 4 e 5) (Tabela 4). 

**Tabela 4:** Razões de chances (*Odds ratio*, OR) e intervalos de confiança de 95% (IC95%) ajustadas de fatores de risco à saúde simultâneos entre adolescentes pelos índices socioeconômicos. Brasil, 2019 (n=4.336)

	Simultaneidade dos fatores de risco									
	Modelo 3^a^ OR (IC95%)			Modelo 4^b^ OR (IC95%)				Modelo 5^c^ OR (IC95%)		
	1	2	3-4	1	2	3-4		1	2	3-4
**Efeito fixo**										
**Características individuais**										
**Sexo**										
Masculino	1,00	1,00	1,00	1,00	1,00	1,00		1,00	1,00	1,00
Feminino	0,84 (0,72; 1,00)	0,59 (0,47;0,75)	0,45 (0,33;0,63)	0,84 (0,72; 0,99)	0,59 (0,46; 0,74)	0,44 (0,32; 0,60)		0,84 (0,71; 0,99)	0,58 (0,46; 0,74)	0,43 (0,31; 0,59)
**Idade** (anos)										
15-17	1,00	1,00	1,00	1,00	1,00	1,00		1,00	1,00	1,00
18-19	0,96 (0,81; 1,14)	1,24 (1,00; 1,56)	2,72 (1,80; 4,13)	0,96 (0,81; 1,14)	1,24 (0,99; 1,55)	2,66 (1,76; 4,01)		0,96 (0,81; 1,14)	1,25 (1,00; 1,55)	2,64 (1,74; 4,00)
**Escolaridade**										
Analfabeto	1,00	1,00	1,00	1,00	1,00	1,00		1,00	1,00	1,00
Fundamental incompleto	2,24 (1,16; 4,33)	7,22 (2,01; 25,10)	1,09 (0,33; 3,58)	2,23 (1,15; 4,35)	7,30 (2,01; 26,53)	1,07 (0,33; 3,42)		2,24 (1,15; 4,36)	7,37 (2,00; 27,1)	1,28 (0,35; 4,63)
Médio incompleto	2,81 (1,40; 5,65)	8,11 (2,41; 27,26)	0,62 (0,20; 1,91)	2,80 (1,39; 5,65)	8,29 (2,45; 28,09)	0,63 (0,21; 1,93)		2,81 (1,39; 5,69)	8,25 (2,40; 28,32)	0,74 (0,22; 2,47)
Superior incompleto	2,00 (1,02; 3,91)	5,31 (1,50; 18,72)	0,41 (0,14; 1,19)	1,98 (1,00; 3,92)	5,53 (1,55; 19,68)	0,43 (0,16; 1,19)		2,44 (0,38; 15,7)	5,45 (1,52; 19,61)	0,51 (0,16; 1,62)
Superior completo	2,41 (0,39; 15,13)	0,84 (0,05; 13,76)	0,80 (0,09; 7,00)	2,42 (0,37; 15,83)	0,93 (0,06; 15,02)	0,88 (0,11; 6,92)		0,91 (0,76; 1,1)	0,91 (0,06; 15,0)	1,10 (0,11; 11,00)
**Estado civil**										
Com companheiro	1,00	1,00	1,00	1,00	1,00	1,00		1,00	1,00	1,00
Sem companheiro	1,11 (0,65; 1,91)	1,60 (0,78; 3,26)	3,77 (1,06; 13,37)	0,92 (0,77; 1,11)	0,72 (0,57; 0,92)	0,49 (0,32; 0,75)		1,12 (0,65; 1,93)	0,74 (0,59; 0,93)	0,54 (0,35; 0,82)
**Zona**										
Urbana	1,00	1,00	1,00	1,00	1,00	1,00		1,00	1,00	1,00
Rural	0,91 (0,76; 1,10)	0,74 (0,58; 0,93)	0,52 (0,34; 0,81)	1,12 (0,65; 1,92)	1,65 (0,81; 3,36)	3,87 (1,10; 13,55)		0,72 (0,50; 1,05)	1,68 (0,83; 3,41)	3,86 (1,09; 13,69)
**Características contextuais**										
**Índice de Desenvolvimento Humano Municipal**										
Médio	1,00	1,00	1,00							
Alto	1,39 (1,10; 1,76)	1,90 (1,63; 2,22)	1,87 (1,03; 3,40)							
Muito alto	2,45 (2,00; 3,01)	4,18 (3,36; 5,20)	6,83 (3,39; 13,75)							
**Índice de Vulnerabilidade Social**										
Muito baixo				1,00	1,00	1,00				
Baixo				0,80 (0,57; 1,11)	0,79 (0,52; 1,21)	0,45 (0,22; 0,96)				
Médio				0,62 (0,43; 0,90)	0,48 (0,31; 0,75)	0,25 (0,12; 0,54)				
**Índice de Gini**										
1º tercil							1,00		1,00	1,00
2º tercil								0,73 (0,54; 0,98)	0,54 (0,35; 0,83)	0,28 (0,15; 0,51)
3º tercil								0,84 (0,71; 0,99)	0,52 (0,38; 0,70)	0,34 (0,19; 0,60)
**Efeito aleatório** (Estado)										
Variância (IC95%)	0,04 (0,01; 0,13)			0,11 (0,07; 0,18)				0,10 (0,05; 0,18)		
Coeficiente de partição de variância (%)	75,16			31,68				39,75		
Coeficiente de correlação intraclasse	0,012			0,032				0,029		
Critério de informação de Akaike	10.249,91			10.276,1				10.253,56		

^a^ Modelo 3: modelo 2 ajustado pelo Índice de Desenvolvimento Humano Municipal; ^b^Modelo 4: modelo 2 ajustado pelo Índice de Vulnerabilidade Social; ^c^Modelo 5: modelo 2 ajustado pelo Índice de Gini.

Com a introdução das variáveis contextuais, a variância diminuiu de 0,16 para 0,04 (Modelo 3), 0,11 (Modelo 4) e 0,10 (Modelo 5). A maior redução foi devida ao ajuste do modelo 3 (CPV 75,2%), assim como o menor índice de correlação intraclasse (0,012) e menor critério de informação de Akaike (10.249,91), indicando que este foi o melhor modelo para explicar a variabilidade existente entre os estados brasileiros para a prevalência simultânea dos fatores de risco.

## Discussão

A prevalência de simultaneidade de comportamentos de risco à saúde entre adolescentes foi influenciada por características individuais (sexo masculino, residir em área urbana, maior idade, não ter companheiro e maior escolaridade) e contextuais. Observou-se que estados com maior Índice de Desenvolvimento Humano (IDH) apresentam maior prevalência de múltiplos comportamentos de risco, enquanto maior vulnerabilidade social e desigualdade econômica estão associadas a menores chances de simultaneidade. A variação entre os estados brasileiros foi parcialmente explicada por fatores contextuais, com o IDH demonstrando maior influência, o que evidencia a importância das desigualdades regionais na determinação desses comportamentos.

Apesar de apresentar limitações inerentes ao seu delineamento transversal, ao uso de dados secundários e autorrelatados (viés de informação e subestimação da prevalência), este estudo oferece contribuições relevantes para a compreensão dos comportamentos de risco entre adolescentes brasileiros. A ampla representatividade da amostra e a análise multinível evidenciam a influência de fatores contextuais e fornecem subsídios importantes para políticas públicas voltadas à redução desses comportamentos.

Tais constatações são consistentes com estudos realizados em outros contextos (4,5), que indicaram padrões semelhantes de simultaneidade de comportamentos de risco, particularmente entre adolescentes do sexo masculino e não brancos. Estudo realizado em 140 países (5), identificou uma alta prevalência de comportamentos de risco individuais, como consumo insuficiente de frutas e vegetais e inatividade física. Outro estudo, em análise de 89 países revelou que a simultaneidade de fatores de risco de estilo de vida, incluindo tabagismo e consumo de álcool, foi predominante entre adolescentes do sexo masculino, com diferenças regionais significativas, mas elevada prevalência nas diferentes regiões do globo (22). Esse fenômeno global sugere que a simultaneidade de fatores de risco é uma questão transversal, embora com manifestações diferenciadas de acordo com as condições socioeconômicas e culturais.

Entre os comportamentos de risco isolados mais prevalentes neste estudo, destaca-se o consumo elevado de alimentos ultraprocessados, um padrão alimentar frequentemente observado também em estudos realizados com adolescentes brasileiros (23-25). Na população geral, o consumo destes alimentos tende a diminuir com a idade e aumentar com a escolaridade e a renda (26). Entre adolescentes, esse hábito associa-se fortemente ao tempo excessivo de tela, hábitos alimentares da infância e ao tipo de escola, sendo mais prevalente entre alunos de escolas privadas (24).

A combinação mais frequente de comportamentos de risco observada — consumo de alimentos ultraprocessados e inatividade física — reitera os achados de outras pesquisas no país (27) e reflete um padrão global identificado em outros locais, onde o baixo consumo de frutas e vegetais é comumente combinado com a inatividade física (5). Cabe ressaltar que embora a falta de atividade física seja um problema, há evidências de que os hábitos sedentários em si são ainda mais prejudiciais e estão mais fortemente associados ao consumo de alimentos ultraprocessados (25). 

A simultaneidade de três comportamentos de risco (consumo de alimentos ultraprocessados, inatividade física e uso habitual de álcool) encontrada corrobora com achados no contexto brasileiro (28) e em estudos de amplitude global (22), donde o consumo de álcool se destaca como componente relevante nas combinações de fatores de risco. Embora a simultaneidade de comportamentos de risco em adolescentes apresente baixa prevalência, decrescendo com o aumento de fatores associados, sua manifestação varia conforme condições individuais e contextuais.

Nas zonas rurais há uma menor chance de acumulação de fatores de risco, o que pode ser atribuído ao modo de vida simples do campo (25). Entretanto, apesar de uma dieta mais natural e de um ambiente menos propenso à inatividade física, observa-se um aumento do etilismo e do tabagismo, associados à percepção deficiente de risco, que resulta da limitada oferta de informações. Em contraste, as áreas urbanas apresentam um cenário distinto, em que a maior exposição a alimentos ultraprocessados, o sedentarismo e o ritmo acelerado da vida, favorecem a adoção de hábitos prejudiciais e restringem as opções de escolhas saudáveis (29).

A idade avançada entre adolescentes associa-se a maior simultaneidade de fatores de risco para doenças crônicas, possivelmente devido à maior autonomia e consequente maior vulnerabilidade, com adoção de hábitos menos saudáveis (10,30). Essa relação intensifica-se entre adolescentes com maior escolaridade, que demonstram maior probabilidade de acumular comportamentos de risco, possivelmente devido ao aumento da liberdade social e comportamental (31,32). Contudo, é importante destacar que o efeito da escolaridade sobre o comportamento de risco foi moderado quando ajustado pelo IDH. Esse ajuste sugere que o nível de desenvolvimento socioeconômico pode atenuar os efeitos negativos associados ao aumento da idade e da escolaridade.

Adolescentes sem companheiros, residentes em estados com alto IDH, apresentaram maior probabilidade de acumular fatores de risco. Embora a relação entre hábitos de vida não saudáveis e estado civil entre adolescentes ainda não seja amplamente explorada na literatura, a associação entre estar solteiro e maior exposição a comportamentos de risco é bem estabelecida, especialmente na faixa etária de análise deste estudo (33). 

Há evidências que o desenvolvimento socioeconômico, com melhorias em educação, saúde e infraestrutura, está associado a um controle mais eficaz de doenças crônicas, devido à maior adoção de comportamentos saudáveis pela população, resultando em menores taxas de mortalidade (34). Todavia, os resultados deste estudo apontam que em estados com maior desenvolvimento aumentam as chances de apresentação de simultaneidade de fatores de risco entre os adolescentes com efeito reverso quando analisado sob a perspectiva da vulnerabilidade social, visto que adolescentes que viviam em regiões com maiores Índices de Vulnerabilidade Social e de Gini tiveram menor probabilidade de ocorrência de comportamentos de risco isolados e em similaridade. 

Convém destacar que os resultados deste estudo convergem com os achados da Pesquisa Nacional de Saúde Escolar 2015 (27), que identificou que o acúmulo de fatores de risco entre adolescentes foi maior entre moradores de cidades em áreas urbanas mais desenvolvidas do país. Isso sugere que comportamentos de risco nesta população tendem a se acentuar em áreas de maior desenvolvimento socioeconômico, nas quais o acúmulo de capital está frequentemente associado a comportamentos prejudiciais à saúde (31). Países da América Latina e Caribe com maior Produto Interno Bruto e IDH, por exemplo, apresentam maior prevalência de sobrepeso e obesidade entre adolescentes (35). Da mesma forma, áreas mais desenvolvidas estão relacionadas à maior prevalência de hipertensão arterial, uma condição amplamente influenciada por hábitos como o consumo elevado de alimentos ultraprocessados, sedentarismo e consumo de álcool e tabaco (36,37).

Embora a vulnerabilidade social esteja geralmente associada a piores indicadores de saúde, devido à dificuldade de acesso a recursos essenciais (38), a relação com o acúmulo de fatores de risco entre adolescentes apresenta uma dinâmica específica e, os resultados deste estudo sugerem que regiões menos desenvolvidas podem atuar como fatores de proteção contra o acúmulo desses comportamentos. Esse efeito precisa ser melhor explorado, mas pode ser explicado por diversos mecanismos sociais e culturais presentes nesses contextos, como a coesão comunitária, o apoio familiar e valores culturais que tendem a mitigar a prevalência de comportamentos de risco (39).

Por fim evidenciamos a influência de variáveis individuais na concorrência de fatores de risco presentes em adolescentes brasileiros, sugerindo que as chances destes adolescentes acumularem fatores de risco variam quando ajustadas por variáveis contextuais e aumentam com o maior desenvolvimento da região em que vivem. Esses resultados reforçam a necessidade de políticas abrangentes de promoção da saúde e bem-estar social, capazes de enfrentar múltiplos fatores de risco às doenças crônicas não transmissíveis entre adolescentes em diferentes regiões do país. Estudos futuros podem explorar a complexidade dos fatores multiníveis que afetam esse grupo, incluindo níveis individuais, familiares, socioeconômicos e de mercado.

## Data Availability

O banco de dados e os códigos de análise utilizados na pesquisa estão disponíveis em https://www.ibge.gov.br/estatisticas/sociais/saude/29540-2013-pesquisa-nacional-de-saude.html?edicao=9177&t=microdados.
